# Viral Dynamic Surveillance in COVID-19 Patients: A Cohort Study

**DOI:** 10.1155/2022/1399268

**Published:** 2022-08-19

**Authors:** DeWu Bi, ZhenCheng Chen, ShanQiu Wei, ZhouHua Xie, Ning Zang, LiDa Mo, ZeDuan Liu, YuanLi Wang, LiangLi Cao, FeiJun Zhao, YanRong Lin, YaQin Qin, XiKe Tang, HanZhen Su, JinAi Zhou, JuanYing Liang, ZhenXu Lan, Lü Lin, ShaoYong Xi, XiaoCheng Luo, QiuYing Ma, XiaoFeng Pang, XiaoLu Luo

**Affiliations:** ^1^Affiliated Infectious Disease Hospital of Nanning, Guangxi Medical University, China; ^2^The Fourth People's Hospital of Nanning, China; ^3^School of Life and Environmental Sciences, Guilin University of Electronic Technology, China; ^4^Guangxi Medical Research Center, Guangxi Medical University, China

## Abstract

**Background:**

Coronavirus disease 2019 (COVID-19) is a potentially fatal pneumonia caused by severe acute respiratory syndrome coronavirus 2 (SARS-CoV-2), especially those of novel SARS-CoV-2 variants and infection has affected over 700 million people globally.

**Methods:**

This retrospective, descriptive study included 118 patients admitted with SARS-CoV-2 infection as confirmed by real-time reverse transcription polymerase chain reaction.

**Results:**

The median duration of detectable SARS-CoV-2 infection in patients with high ALT, AST, and PLT/LYMPH, or low CD4+, CD8+, and PLT/MONO was considerably longer. In the risk factor model, multivariate analysis was performed for the estimation of ALT (HR, 0.54; 95% CI, 0.36-0.81), AST (HR, 0.56; 95% CI, 0.34-0.93), CD4+ (HR,0.77; 95% CI, 0.48-1.24), CD8+ (HR,0.64; 95% CI, 0.37-1.11), PLT/LYMPH (HR, 1.16; 95% CI, 0.76-1.77), and PLT/MONO (HR, 0.64; 95% CI, 0.43-0.94).

**Conclusions:**

The longer viral RNA duration was associated with a higher International Prognostic Index score (*p* = 0.0013), demonstrating for the first time that multivariate features of the bioindicators closely associated with SARS-CoV-2-infected patients clear the virus.

## 1. Introduction

First detected in December 2019, a highly contagious pneumonia, called novel coronavirus disease 2019 (COVID-19), has spread worldwide and caused a global public health crisis [1, 2]. The World Health Organization (WHO) reported more than 248 million confirmed cases of COVID-19, which have resulted in more than five million fatalities globally as of November 3, 2021 [3]. The causative agent was identified as severe acute respiratory syndrome coronavirus 2 (SARS-CoV-2), a novel beta-coronavirus, in bronchoalveolar lavage fluid samples from patients [1]. SARS-CoV-2 is distinct from severe acute respiratory syndrome coronavirus (SARS-CoV), which arose in Guangdong, China, in 2002 [4], and led to more than 8,000 infections and 700 deaths globally from 2002 to 2003 [5], and Middle East respiratory coronavirus (MERS-CoV), which was first reported in Saudi Arabia in 2012 [6], causing infection-related mortality rates of 41% and 35% in Saudi Arabia and globally, respectively [7]. SARS-CoV-2 belongs to the orthocoronavirinae subfamily; its highest genetic similarity is to a bat coronavirus, Bat CoV RaTG13, with which it is 96% identical at the whole-genome level [8, 9]. Further investigation is needed for definitive determination of the origin of SARS-CoV-2.

COVID-19, which has a clinical profile like severe acute respiratory syndrome (SARS), can result in death from underlying disease or acute respiratory distress syndrome due to lung injury [1–2, 4–5, 8]. Early evidence linked the spread to seafood markets, but human-to-human transmission has now been confirmed [2, 10]. Characterization of the high-affinity interaction between SARS-CoV-2 and angiotensin-converting enzyme-2 (ACE2), a cellular receptor for SARS-CoV and SARS-CoV-2, revealed that it enables efficient transmission by direct contact or via respiratory droplets [11, 12]. The presence of inframicrobes in faeces and blood has been confirmed via polymerase chain reaction (PCR) [13], indicating the danger of aerosol generation and propagation of COVID-19 in small, enclosed spaces.

Notably, different SARS-CoV-2 variants defining nonsynonymous mutations have successively emerged worldwide [14–18]. SARS-CoV-2 is one of the deadliest viruses that infect both humans and animals including cats [19, 20], dogs [20, 21], and mink [22], then infected animals can transmit the virus back to humans [21, 22]. Although the mechanism of animal-to human transmission is unclear, evidence suggests that these variants exhibit superior cross-species transmission capacity. Compared with the wild-type strain Wuhan-Hu-1, recently identified variants B.1.351 (501Y.V2) in South Africa [23], B.1.1.7 (VUI-202012/01) in the United Kingdom [24], B.1.1.28.1 (P.1) in Brazil [16], B.1.429 (CAL.20C) in Southern California [17, 25], and B.1.617 in India [18] exhibit robust transmissibility owing to residue changed of these variants. These mutations, contributing to stabilize the complex structure of spike protein with ACE2 [26] and be responsible for SARS-CoV-2 immune escape [27], was the worldwide spread of a pandemic.

## 2. Materials and Methods

### 2.1. Study Design and Patients

This retrospective, descriptive study was approved by the ethics committee of The Fourth People's Hospital of Nanning (Affiliated Infectious Disease Hospital of Nanning, Guangxi Medical University). The hospital is responsible for the treatment of patients with COVID-19, as assigned by the government. This case series was approved by The Ethics Committee of The Fourth People's Hospital of Nanning (nos. [2020]05, [2020]2, [2020]3, and [2020]4). All data of patients who had confirmed SARS-CoV-2 infection and diagnosed according to the WHO interim guidance at The Fourth People's Hospital of Nanning from January 21, 2020, to October 21, 2021, were obtained via hospital electronic medical records. The final laboratory diagnosis of COVID-19 was completed by the Nanning Center for Disease Control and Prevention.

### 2.2. Procedures

Throat swabs, sputum, faeces, urine, semen, vaginal discharge, and milk specimens were collected from patients. RNA was extracted from the samples using the Nucleic Acid Isolation Kit (Magnetic Beads) (SDK60105; Jiangsu, China), according to the manufacturer's protocol. The RNA was retained after removing human nucleic acids. We detected SARS-CoV-2 using primers and probes for two envelope genes of interest, open reading frame 1ab (Orf1ab), and nucleocapsid protein (N). The nucleic acid amplification reaction buffer, enzyme mixture, Orf1ab/N reaction solution, and RNA suspension were placed into a centrifuge tube. In addition, positive and negative controls were included to verify diagnostic reliability. The real-time reverse transcription PCR (RT-PCR) assay was performed using a SARS-CoV-2 nucleic acid detection kit according to the instructions provided by Guangzhou Daan Biotechnology Co, Ltd. The RT-PCR amplification protocol comprised reverse transcription at 50°C for 30 minutes and 95°C for 5 minutes, followed by 45 cycles of denaturation at 95°C for 10 seconds, and annealing, extending, and fluorescent signal detection at 55°C for 40 seconds.

The RT-PCR assay was considered negative if the cycle threshold value (Ct-value) was >38 and positive if the Ct-value was ≤35. Samples were retested if the Ct-value was >35 and ≤38. The results were considered positive if the Ct-value remained between 35 and 38 after retesting.

### 2.3. Data Collection

We reviewed the data related to the epidemiological, clinical, laboratory, and radiological characteristics; treatment; and outcome of the patients via detail collection forms using their electronic medical records. The patient information, which included demographics, epidemiological data, comorbidities, symptoms, signs, laboratory results, chest computed tomography findings, and treatment measures, was collected and analyzed after disease onset.

### 2.4. Statistical Analysis

Continuous variables are presented as mean and standard deviation (SD), if normally distributed, or median and 95% confidence interval (95% CI), if not. Categorical variables are presented as count (%). We compared the means of continuous variables using independent group *t*-tests when the data were normally distributed. Otherwise, we used the Mann–Whitney test. The proportions of categorical variables were compared using the *χ*^2^ test. Kaplan-Meier analysis was performed using the Gehan-Breslow-Wilcoxon test. Statistical analyses were conducted using GraphPad Prism software (version 8·0). A *p* value less than 0.05 (<0.05) was considered significant.

## 3. Results

### 3.1. Viral Duration

#### 3.1.1. Correlation between Viral Duration in Different Disease Severity

**Figure 1:**
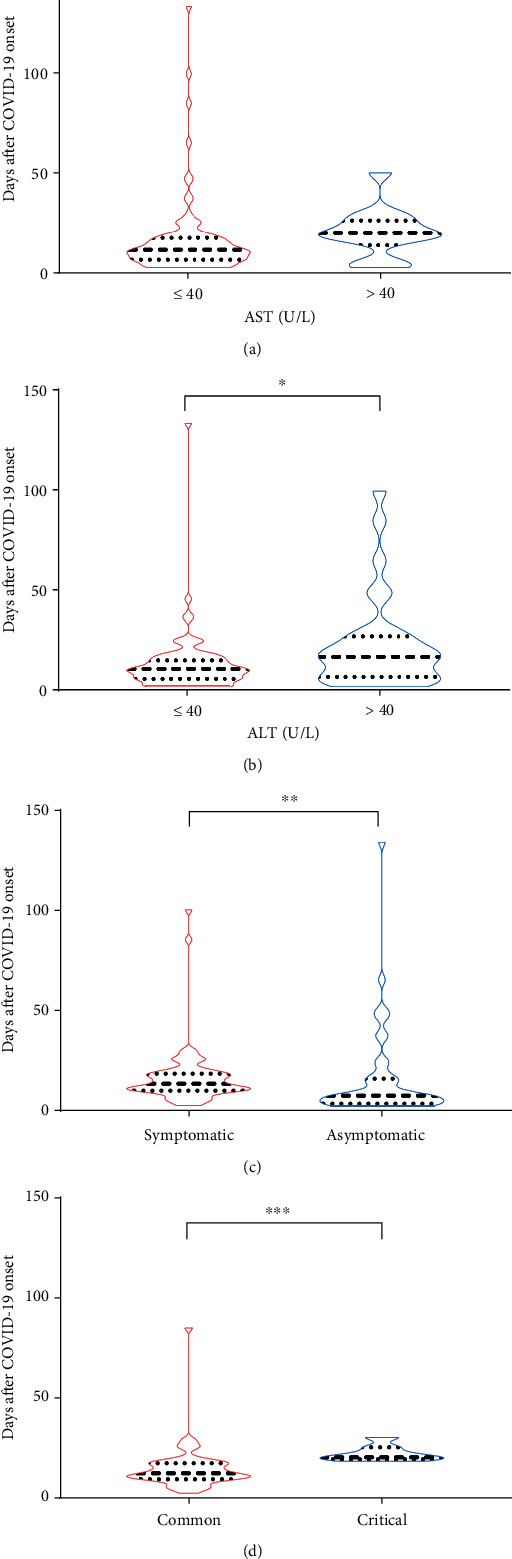
Duration of SARS-CoV-2 infection analyzed by AST, ALT, clinical features, and disease severity. (a) The duration of different viral shedding for different aspartate aminotransferase level. (b) The duration of different viral shedding for different alanine aminotransferase level. (c) Duration of viral diversification across clinical characteristics. (d) The duration of viral divergence varies among disease severity.

At admission, the median duration of SARS-CoV-2 in patients with critical disease (median duration of virus: 20 days (95% CI: 18–30)) was significantly longer than in patients with common disease (median duration of virus: 12 days (95% CI: 11–15), *p* = 0 · 0003) (Figure 1(d)). Similarly, the median duration of SARS-CoV-2 in symptomatic patients (median duration of virus: 13 days (95% CI: 11–17)) was significantly longer than in asymptomatic patients (median duration of virus: 7 days (95% CI: 5–11), *p* = 0 · 0019) (Figure 1(c)).

Of note, patients with higher alanine aminotransferase (median duration of virus: 17 days [95% CI: 9–24]) had a significantly longer duration of SARS-CoV-2 infection than patients with normal levels (median duration of virus: 11 days (95% CI: 9–12), *p* = 0 · 015) (Figure 1(b)). Similarly, patients who had higher aspartate aminotransferase (median duration of virus: 20 days (95% CI: 5–28)) tended to have a significantly longer duration of SARS-CoV-2 infection than those with normal levels (median duration of virus: 11 days (95% CI: 9–13), *p* = 0 · 0197) (Figure 1(a)).

#### 3.1.2. Factors Associated with Duration of Virus

**Figure 2:**
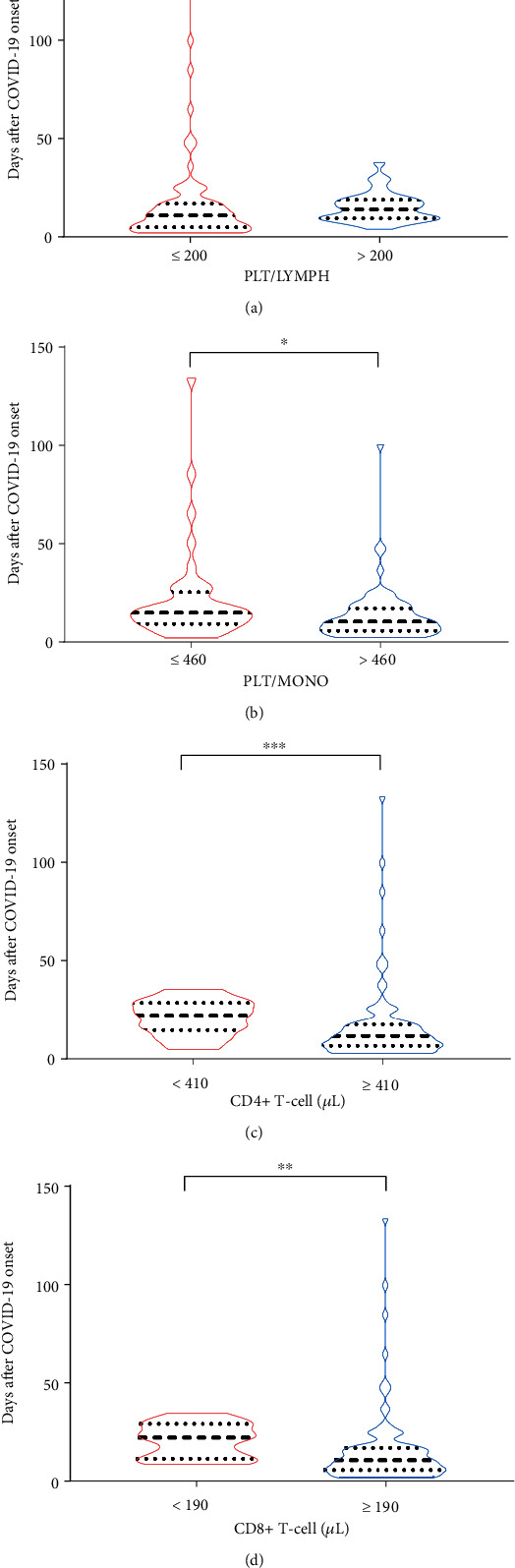
Comparison of the duration of SARS-CoV-2 among groups with varying PLT/LYMPH, PLT/MONO, CD4+, and CD8+ T-cell levels. (a) Viral duration differ for different PLT/LYMPH ratio. (b) Viral duration differs for different PLT/MONO ratio. (c) Viral duration differs for different CD4+ T-cell level. (d) Viral duration differs for different CD8+ T-cell level. Standard normal value for PLT/LYMPH, PLT/MONO, and CD4+/CD8+ are [94, 200], [20, 100], and [1, 2], respectively.

Patients with lower CD4+ T-cell counts had a significantly longer duration of SARS-CoV-2 infection than patients with higher counts (median duration of virus: 22 days (95% CI: 15–28) and 11 days (95% CI: 8–13), respectively; *p* = 0 · 0009) (Figure 2(c)). Similarly, patients who had higher CD8+ T-cell counts tended to have a significantly shorter duration of SARS-CoV-2 infection than those with lower CD8+ T-cell counts (median duration of virus: 11 days (95% CI: 9–14) and 23 days (95% CI: 11–30), respectively; *p* = 0 · 0063) (Figure 2(d)).

Among the patients, SARS-CoV-2 persisted markedly longer in patients with PLT/LYMPH ratio > 200 than in those ≤200 (median duration of virus: 14 days (95% CI: 10–18) and 11 days (95% CI: 7–13), respectively; *p* = 0 · 045) (Figure 2(a)). The duration of SARS-CoV-2 infection was longer in patients with PLT/MONO ratio ≤ 460 (median duration of virus: 15 days (95% CI: 11–17)) than in those with a ratio > 460 (median duration of virus: 10 days (95% CI: 7–12), *p* = 0 · 0161) (Figure 2(b)).

### 3.2. Survival Analysis

#### 3.2.1. Kaplan-Meier and Multivariable Cox Regression Model Analysis

**Figure 3:**
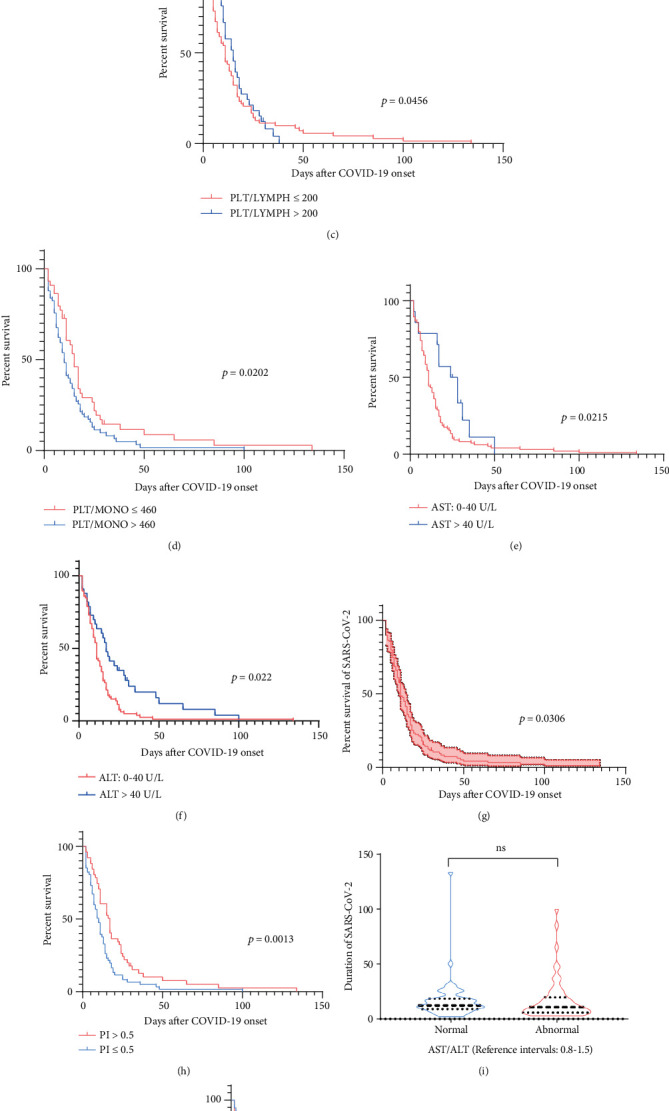
Abnormal biological variables were associated with longer viral duration. (a) Kaplan-Meier curve for CD4+ T cell profiles of patients with low and normal level. (b) Kaplan-Meier curve for CD8+ T cell profiles of patients with low and normal level. (c) Kaplan-Meier curve for PLT/LYMPH of patients with low and high ratio. (d) Kaplan-Meier curve for PLT/MONO of patients with low and high ratio. (e) Kaplan-Meier curve for AST profiles of patients with high and normal level. (f) Kaplan-Meier curve for ALT profiles of patients with high and normal level. (g) Multivariate analysis of the experimental parameters using Cox proportional hazards regression models. (h) Kaplan-Meier curve for PI profiles of patients with high and low value. (i) The duration of different viral shedding for aspartate/alanine amino transferase level. (j) Kaplan-Meier curve for AST/ALT ratio profiles of patients with high and normal level. Standard normal value for AST/ALT ratio is [0.8, 1.5].

Kaplan-Meier curve illustrated the duration of viral RNA shedding of patients with COVID-19 during follow-up (Figures 3(a)–3(f)). The risk of the duration of viral RNA shedding was higher in patients with higher PLT/LYMPH and lower CD4+ T cells (HR, 1.16 (95% CI, 0.76-1.77) vs. 0.77 (95% CI, 0.48-1.24)); lower CD8+ T cell and higher PLT/MONO were found to be increased duration of viral shedding (HR, 0.64 (95% CI, 0.37-1.11) vs. 0.64 (95% CI, 0.43-0.94)). AST and ALT were similar predictive of duration of viral shedding (HR, 0.56 (95% CI, 0.34-0.93) vs. 0.54 (95% CI, 0.36-0.82)). Of note, patients with high PLT/LYMPH ratio had a significantly higher risk than other abnormal biological indices.

Multivariable Cox proportion hazard regression model was performed (Figure 3(g)). Prognostic index (PI) was associated with AST (RR, 0.97; 95% CI, 0.94-0.99), ALT (RR, 1.01; 95% CI, 0.99-1.02), CD4+ (RR, 1.00; 95% CI, 0.99-1.01), CD8+ (RR, 0.99; 95% CI, 0.99-1.00), PLT/LYMPH (RR, 0.99; 95% CI, 0.99-1.00), and PLT/MONO (RR,1.00; 95% CI, 0.99-1.00). Prognostic index was shown as a function of the number of risks at diagnosis and therapy. Indeed, we conformed that patients with high PI had a longer duration of viral shedding than those with low PI (Figure 3(h)). Therefore, despite the fact that these regular clinical examination parameters were obtained, the features and underlying the parameters were consistently and closely associated with adverse prognosis in patients with COVID-19. Hence, we have identified and validated a function capable of predicting the duration of viral shedding in patients with COVID-19.

In conclusion, we proposed a multivariable Cox proportion hazard regression model, where the prognostic index resulted from a combination of factors. Collectively, these parameters suggest the duration of viral shedding and disease progression.

(1) $$\begin{array}{c} \text{PI}=h\left(t,x\right)=h_{0}\left(\text{t}\right)\exp\left(0.005∙\text{ALT}-0.03∙\text{AST}+0.0005∙{\text{CD}}_{4}^{+}-0.0008∙{\text{CD}}_{8}^{+}-0.001∙\frac{\text{PLT}}{\text{LYMPH}}+0.0005∙\frac{\text{PLT}}{\text{MONO}}\right)\left(\chi^{2}=13.91;\text{DF}=6;p=0.03\right). \end{array}$$

## 4. Discussion

In the present study, we systematically compared the dynamics of SARS-CoV-2 infection and disease progression via 118 patients to characterize the factors that influence host defense against SARS-CoV-2.

Previous studies showed that cellular immune response, in addition to the humoral immune response, plays an important role in T-cell immunity-mediated virus clearance [28–30]. We further investigated the kinetic determinants of virus shedding and found that higher frequencies of CD4+ and CD8+ T-cells may be associated with better virus clearance. Clinical strategies to improve the outcomes of patients with COVID-19 who receive antiviral therapy should consider their CD8+ T-cell counts. Our findings suggest that CD4+ and CD8+ T-cells play an important role in restricting virus replication and suppressing the progression of COVID-19 and can be used to assess prognosis. Lower CD4+ and CD8+ T-cell levels were associated with an increased risk of a worse clearance of the virus.

Platelets showed important and distinct characteristics in bacterial [31], malaria [32], and virus [33] infection. Large scale clinical studies in many countries and centers suggested that thrombocytopenia was significantly associated with poor prognosis, but the mechanism was unknown [34]. We found no difference in the duration of viral shedding in those platelets less than 100 × 10^9^/L vs. those platelets 100 × 10^9^/L and more, however, we also conducted prespecified secondary analysis to elucidate the platelets were associated with risks potentially related to viral clearance. Higher PLT/LYMPH and lower PLT/MONO ratio were associated with an increased risk of elongation time for viral shedding. Six of the risk factors seemed to have much impact on the duration of viral RNA shedding, however, PLT/LYMPH and PLT/MONO ratio were two significantly high-risk factors, which may be attributed to the fact that platelets owned various immunological functions and exerted its regulatory role on the lymphocytes and monocytic cells.

Most patients with COVID-19 suffer from liver dysfunction [35], and we observed that low levels of alanine aminotransferase and aspartate aminotransferase correlate with a short duration of SARS-CoV-2 infection. Therefore, alanine aminotransferase and aspartate aminotransferase levels may be a prognostic marker in patients with COVID-19.

This study was the first to explore the potential prognostic value of these risk factors assessed during COVID-19 pandemic, SARS-CoV-2-infected patients. These risk factors considered together suggested that the prognosis for patients with COVID-19 should be regarded as an accurate prediction by the proportional hazards regression model. Consequently, it is significantly important to group by prognostic index for monitoring the changes in viral dynamics and guiding clinical therapies.

## 5. Patents

This was a retrospective, descriptive study, and no patients were directly involved in the study design, setting, research questions, or outcomes measures. No patients were asked to advise on interpretation or writing up of results. Demographics and baseline characteristics of the 118 patients with COVID-19 were summarized in Table 1.

## Figures and Tables

**Table 1:** Demographics and baseline characteristics of patients with COVID-19.

	All patients (*n* = 118)	Symptomatic (*n* = 70)	Asymptomatic (*n* = 48)	*p* value
Age, years				
Mean (IQR)	38 (30-49)	43 (34-57)	33 (27-44)	0·003
Range	85	85	53	
Sex				
Female	37 (31%)	31 (45%)	6 (13%)	<0·001
Male	81 (69%)	39 (55%)	42 (87%)	
Races				
Han	102	58	44	0.2733
Other	16	12	4	
Occupation				
Civil servant	3 (3%)	3 (4%)	0 (0%)	0·121
Farmer	23 (19%)	12 (17%)	11 (23%)	0·376
Worker	6 (5%)	4 (6%)	2 (4%)	0.747
Self-employed	8 (7%)	6 (9%)	2 (4%)	0·250
Employee	13 (11%)	10 (14%)	3 (6%)	0·097
Student	9 (8%)	6 (9%)	3 (6%)	0·592
Retired	10 (8%)	9 (13%)	1 (2%)	0·005
Medical staff	3 (3%)	3 (4%)	0 (0%)	0·121
Other	43 (36%)	17 (24%)	26 (55%)	<0·001
Comorbidities^⸸^				
Diabetes	5 (4%)	4 (6%)	1 (2%)	0·279
Hypertension	17 (14%)	11 (16%)	6 (13%)	0·688
Hyperlipidaemia	16 (13%)	15 (21%)	1 (2%)	<0·001
Liver dysfunction	20 (17%)	14 (20%)	6 (13%)	0·252
Gastrointestinal complaints	5 (4%)	4 (6%)	1 (2%)	0·279
Bile duct stones	4 (3%)	3 (4%)	1 (2%)	0·682
Cardiac/cerebrovascular diseases	6 (5%)	4 (6%)	2 (4%)	0·747
Kidney disease	4 (3%)	4 (6%)	0 (0%)	0·028
Respiratory failure	2 (2%)	2 (3%)	0 (0%)	0·246
Anaemia	8 (7%)	8 (11%)	0 (0%)	<0·001
Electrolyte disturbance	10 (8%)	7 (10%)	3 (6%)	0.435
Bacterial pneumonia	11 (9%)	11 (16%)	0 (0%)	<0·001
Pulmonary tuberculosis	3 (2%)	3 (4%)	1 (2%)	0·682
Hepatitis B virus	4 (3%)	2 (3%)	2 (4%)	>0·999

Data are *n* (%). ^⸸^Some patients had multiple comorbidities. IQR is interquartile range.

## Data Availability

Epidemiological, clinical, and laboratory characteristics and treatment and outcome data were obtained through data collection forms from hospital electronic medical records.

## Abbreviations

AST: Aspartate aminotransferase
ALT: Alanine aminotransferase
PLT/LYMPH ratio: Platelet to lymphocyte ratio
PLT/MONO: Platelet to monocyte ratio
PI: Prognostic index.
